# Neural Precursor Cells Expanded Inside the 3D Micro-Scaffold Nichoid Present Different Non-Coding RNAs Profiles and Transcript Isoforms Expression: Possible Epigenetic Modulation by 3D Growth

**DOI:** 10.3390/biomedicines9091120

**Published:** 2021-08-31

**Authors:** Letizia Messa, Bianca Barzaghini, Federica Rey, Cecilia Pandini, Gian Vincenzo Zuccotti, Cristina Cereda, Stephana Carelli, Manuela Teresa Raimondi

**Affiliations:** 1 Department of Chemistry, Materials and Chemical Engineering “Giulio Natta”, Politecnico di Milano, 20157 Milan, Italy; letizia.messa@mail.polimi.it (L.M.); bianca.barzaghini@polimi.it (B.B.); manuela.raimondi@polimi.it (M.T.R.); 2 Department of Biomedical and Clinical Sciences “L. Sacco”, University of Milan, Via Grassi 74, 20157 Milan, Italy; federica.rey@unimi.it (F.R.); gianvincenzo.zuccotti@unimi.it (G.V.Z.); 3 Pediatric Clinical Research Center Fondazione “Romeo ed Enrica Invernizzi”, University of Milano, 20157 Milan, Italy; 4 Genomic and Post-Genomic Center, Istituto di Ricerca e Cura a Carattere Scientifico Mondino Foundation, 27100 Pavia, Italy; cecilia.pandini@unimi.it (C.P.); cristina.cereda@asst-fbf-sacco.it (C.C.); 5 Department of Biology and Biotechnology “L. Spallanzani”, University of Pavia, 27100 Pavia, Italy; 6 Department of Pediatrics, Children’s Hospital “V. Buzzi”, 20157 Milan, Italy

**Keywords:** Nichoid, RNA-seq, non-coding RNAs, RNA interactions, mechanotransduction, 3D-microscaffold, alternative splicing, isoform switching

## Abstract

Non-coding RNAs show relevant implications in various biological and pathological processes. Thus, understanding the biological implications of these molecules in stem cell biology still represents a major challenge. The aim of this work is to study the transcriptional dysregulation of 357 non-coding genes, found through RNA-Seq approach, in murine neural precursor cells expanded inside the 3D micro-scaffold Nichoid versus standard culture conditions. Through weighted co-expression network analysis and functional enrichment, we highlight the role of non-coding RNAs in altering the expression of coding genes involved in mechanotransduction, stemness, and neural differentiation. Moreover, as non-coding RNAs are poorly conserved between species, we focus on those with human homologue sequences, performing further computational characterization. Lastly, we looked for isoform switching as possible mechanism in altering coding and non-coding gene expression. Our results provide a comprehensive dissection of the 3D scaffold Nichoid’s influence on the biological and genetic response of neural precursor cells. These findings shed light on the possible role of non-coding RNAs in 3D cell growth, indicating that also non-coding RNAs are implicated in cellular response to mechanical stimuli.

## 1. Introduction

In the entire human genome, only 1–2% of the codes are composed of proteins, while the remaining percentage is composed of untranslated sequences previously classified as “junk DNA” [1,2]. This definition could not be farther from the truth, as in recent years these regions have been found to code for numerous transcription regulators, with a specific role for non-coding RNAs. Specifically, RNAs could be divided into coding RNAs, with transcripts that are translated into peptides and thus code for a protein product, and non-coding RNAs, that do not code for a specific protein, but are implicated in numerous biological and pathological processes [3,4,5,6,7,8]. Even with significant new evidence arising each year, understanding the biological functions of these molecules still represents one of the major challenges in both molecular and cell biology [3,4,5]. Specifically, non-coding RNAs can be sub-divided into small non-coding RNAs, smaller than 200 bp, and long non-coding (lncRNAs) longer than 200 bp. Small non-coding RNAs include small nuclear RNAs (snRNAs), small nucleolar RNAs (snoRNAs), microRNAs (miRNAs), and piwi-interacting RNAs (piRNAs). LncRNAs include rRNA (ribosomal RNA), mRNA-like intergenic transcripts (lincRNAs), sense intronic RNAs, and lncRNAs that share a bidirectional promoter and natural antisense transcripts (NAT). Non-coding RNAs are involved at different levels of gene expression in physiology and development, including chromatin architecture, epigenetic memory, transcription, RNA splicing, editing, translation, and turnover [2,4,9]. Indeed, multiple evidence suggests that they could work as signals, scaffolds for protein–protein interactions, molecular decoys, and guides to target elements in the genome or transcriptome [10].

Recently, non-coding genes have been found to be implicated in stemness and differentiation [11]. Specifically, stemness-related genes can be influenced by lncRNAs either at a transcriptional level or through epigenetic regulation of their expression [12]. Indeed, they can enhance transcription through binding of genes promoters, or actively bind to transcriptional factors in gene regulation [12]. Particular relevance goes to Neural Stem Cells (NSCs), where non-coding genes have been found to be involved in important functions [11,13,14,15,16] such as stemness maintenance and differentiation [11]. Furthermore, small non-coding RNAs have been implicated in these processes, and these include *miR-342-5p*, a downstream effector of Notch signaling which regulates the differentiation of NSCs and intermediate neural progenitors into astrocytes [13]. A strong implication is also present for lncRNAs as in the adult brain and in embryonic stem cells several lncRNAs were found to be specifically deregulated, suggesting a potential role in central nervous system development [17]. Other regulators include *PSORS1C3*, a lncRNA overlapped with *OCT4* which is a novel fine-tuner of its expression in non-pluripotent cells [14], and *TUNA*, detected at the promoters of *Nanog, Sox2*, and *Fgf4*, required for pluripotency and neural differentiation [18]. Furthermore, we also reported in our previous work the implications of a specific panel of lncRNAs, which are dysregulated during murine NSCs differentiation and exert their action through the interaction with the RNA binding protein *ELAVL1 (HuR)* [11].

In recent years, growing efforts in the field of biomedical research were directed towards finding new strategies to mimic the physiological environment ex vivo, in order to better simulate the native stemness potential of the cells, recreating a niche-like environment [19,20,21,22,23]. Here, we focused on the potential of the scaffold called “Nichoid” [24,25], engineered in the form of a 3D micro-grid in order to mimic the 3D microscopic architecture of a stem cells niche. The Nichoid already demonstrated able to maintain the stemness capacity of primary rat mesenchymal stem cells (MSCs), human bone marrow-derived MSCs, mouse ES cells [26,27,28,29] and NPCs [30,31].

In this context, we have already investigated how the 3D spatial structure of the Nichoid scaffold can influence gene expression through the alteration of mechanotransduction-related processes, subsequently influencing the stemness and therapeutic potentials of NPCs [30,31,32]. Specifically, the deregulated coding genes and subsequent pathways analysis showed that the Nichoid causes alterations in genes starting at a membrane level, through an impact on signaling transduction and cellular metabolism, which ultimately leads to altered nuclear activity and thus changes in cellular functions. These pathways include focal adhesions, integrins, cadherins, and Rho activation, fundamental in bundling of actin filaments and promoting contractility. Furthermore, significant pathways as MAPK cascade, PI3K-Akt, AMPK, Wnt, and RAP1 signaling, implicated in proliferation, cell motility, and migration result deregulated in NPCs grown inside the Nichoid respect to the control condition, all leading to a final coral alteration in coding genes expression [32].

Little is known on the role of non-coding genes in alterations due to a 3D micro-scaffold cells expansion. In 2017, a group of researchers investigated how the stiffness of the matrix regulates the transcriptome profile of human aortic and coronary vascular smooth muscle cells (VSMCs) and identified potential key lncRNAs regulators [33]. Furthermore, the same authors reported that *MALAT1* is a positive regulator of VSMCs proliferation and migration in response to ECM stiffness [31].

We thus investigated the transcriptional deregulation of non-coding RNAs present in NPCs grown in standard floating conditions or inside the Nichoid through RNA-Sequencing (RNA-Seq) approach [32], performing a computational dissection and highlighting their co-interactions with coding genes and potential pathways implications, with a focus on mechanotransduction and stemness.

## 2. Materials and Methods

### 2.1. Nichoids Microfabrication

Nichoids were fabricated on 12 mm of diameter glass coverslips with a direct laser writing technique, known as two photon laser polymerization (2PP) that guarantees a computer-designed 3D structure with a spatial resolution down to 100 nm. Nichoids were obtained through polymerizing a biocompatible photoresist termed SZ2080, extensively validated for stem cells cultures, chemically inert and with a Young’s modulus around 0.14 GPa [28,34]. Specifically, zirconium propoxide (Sigma-Aldrich, St. Louis, MO, USA) and methacryloxypropil trimethoxysilane (Sigma-Aldrich, St. Louis, MO, USA) formed a sol–gel-synthetized silicon–zirconium hybrid inorganic–organic resin. Moreover, 1% concentration of Irg photoinitiator (Irgacure 369, 2-Benzyl-2-dimethylamino-1-(4-morpholinophenyl)-butanone-1) allowed to start the photopolymerization process [34]. SZ2080 offers biocompatibility, good optical transmission, chemical and electrochemical inertia, and long-term stability [32].

After the fabrication process, samples were developed in a solution of 50% (*v*/*v*) methyl isobutyl ketone and 50% (*v*/*v*) isopropyl alcohol solution (Sigma-Aldrich, St. Louis, MO, USA) [28,35,36]. The use of 2PP allowed the creation of a 3D structure with a precise geometry at cellular level. Specifically, the elementary unit of the Nichoid is a single niche of 90 × 90 μm and 30 μm height [33,37]. A total of 25 single niches (5 × 5 niches) make up a single Nichoid block. Nichoid blocks were fabricated to cover up 8 mm of the coverslips surface with a constant spacing of 15 μm.

### 2.2. Substrate Preparation

Nichoids were washed for 90 min in 70% ethanol (VWR), rinsed repeatedly in sterile deionized water, and dried under UV light for 90 min in sterile conditions.

### 2.3. Primary Cells Isolation and Culture

NPCs expressing green fluorescent protein (GFP) were isolated 6 h post-mortem from adult C57BL/6-Tg (UBC-GFP) 30Scha/J mice weighing 25–30 g (Charles River) as previously described [38,39,40,41,42]. All animals’ procedures conform to the European Communities Directive of September 2010 (2010/63/UE) and have been approved by the Review Committee of the University of Milan. The specific codes are N° 778/2017; 535/2017. NPCs were maintained in culture in Neurobasal Medium (GIBCO™, Life Technologies, Carlsbad, CA, USA) containing 2% B-27 supplement (Life Technologies, Carlsbad, CA, USA), 2% L-Glutamine (Euroclone, Pero (MI), Italy), 1% penicillin and streptomycin (Euroclone, Pero, Italy), b-FGF (human recombinant, 20 ng/mL), and hEGF (human recombinant, 20 ng/mL).

### 2.4. Cells’ Seeding in the Nichoid

NPCs maintained in culture were harvested, collected by centrifugation (123× *g* for 10 min), mechanically dissociated and counted with trypan blue exclusion test (Sigma Aldrich, St. Louis, MO, USA). A total of 10,000 cells were seeded at the center of the Nichoid in a single drop of 35 μL NPCs medium. The multi-well was kept in the incubator for 1 h to allow cells to enter the 3D niches and then 465 μL of NPCs medium were added. Specifically, three experiments were performed each including one replicate of NPCs grown in standard floating conditions for 7 days and one replicate of NPCs grown inside the Nichoid for 7 days.

### 2.5. Environmental Scanning Electron Microscopy (ESEM)

Two Nichoids samples were used, and 6 fields were acquired for each Nichoid (n = 12). ESEM analysis was performed by plating 10 000 NPCs inside the Nichoid and keeping them in standard growth condition for 7 days. Samples were fixed by dehydration with ethanol. More specifically, the culture medium was removed, and the samples were incubated for 2 h at room temperature with a solution composed of 1.5% (*v*/*v*) glutaraldehyde 50% (*v*/*v*) and 0.1 M sodium cacodylate. The samples were rinsed with 0.1 M sodium cacodylate buffer and incubated for 5 min in increasing ethanol concentrations (20–30–40–50–60–70–80–90–96–100% *v*/*v*). This passage was repeated twice. The images were acquired using the ESEM ZEISS EVO 50 EP.

### 2.6. RNA Extraction

Total RNA from cultured cells was isolated using TRIzol Reagent (Invitrogen) in accordance with manufacturer’s instructions. Concentration and quality of the extracted RNA were determined using a spectrophotometer (NANOPhotometer^®^ NP80, IMPLEN, Munich, Germany) and a 2100 Bioanalyzer (Agilent RNA 6000 Nano Kit, Waldbronn, Germany); RNAs with a 260:280 ratio of ≥1.5 and an RNA integrity number of ≥8 were subjected to deep sequencing.

### 2.7. Libraries Preparation for RNA-Seq and Bioinformatic Data Analysis

For RNA-Seq 3 samples per condition were analyzed (N = 3). Sequencing libraries were prepared with TruSeq Stranded Total RNA kit (Illumina, San Diego, CA, USA) using 200 ng total RNA. Qualities of sequencing libraries were assessed with 4200 TapeStation with the DNA1000 reagent kit. RNA processing was carried out using Illumina NextSeq 500 Sequencing. FastQ files were generated via llumina bcl2fastq2 (Version 2.17.1.14—https://support.illumina.com/downloads/bcl2fastq-conversion-software-v2-20.html (accessed on 30 June 2019) starting from raw sequencing reads produced by Illumina NextSeq sequencer. Quality of individual sequences were evaluated using FastQC software (see Code Availability 1) after adapter trimming with the cutadapt software. Reads were computed using the STAR/RSEM software [42] using Gencode Release m24 (GRCm38) as a reference, using the “-strandness forward” option and details of raw reads are reported in Table 1. Differential expression analysis for mRNA was performed using R package DESeq2 [43], selected because of its superior performance in identifying isoforms differential expression [44]. Coding and non-coding genes were considered differentially expressed and retained for further analysis with |log_2_(Nichoid sample/control sample)| ≥ 1 and False Discovery Rate (FDR) ≤ 0.1. This choice is motivated by the decision to maximize the sensitivity of this analysis in order to perform a massive screening and identify candidate genes to be validated with a wider sample population with real-time analysis.

### 2.8. Coding and ncRNAs Co-Expression Analysis

Coding RNAs’ co-expression with ncRNAs was performed using weighted gene co-expression network analysis (WGCNA) R (https://CRAN.R-project.org/package=WGCNA (accessed on 28 January 2021) [45,46,47]. The 100 most deregulated coding and non-coding genes (in terms of |log_2_FoldChange|) in Nichoid samples compared to standard floating conditions were selected for this analysis. The soft thresholding power was chosen considering the criterion of approximate scale-free topology. The WGCNA R package was used to generate and identify a cluster dendrogram with branches corresponding to the gene co-expression modules [48]. Specifically, combined with the topological overlap matrix with the hierarchical average linkage clustering method, the gene modules of each gene network were identified. The overall expression patterns within each module were calculated and displayed as heatmap with correlation values for each module and condition. Modules genes with a Pearson’s correlation coefficient of >|0.5| were considered. The deregulated coding and non-coding genes from the blue and the turquoise modules were used for network visualization through the Cytoscape software (http://www.cytoscape.org/ (accessed on 30 January 2021). Network nodes represent gene expression profiles, while undirected edges values are the pairwise correlations between gene expressions.

### 2.9. Functional Enrichment Analysis

Gene enrichment analysis was performed via g:Profiler web tool (https://biit.cs.ut.ee/gprofiler/gost (accessed on 2 February 2021), ranking genes according to their absolute fold change and using Bonferroni-Hochberg FDR of 0.05 as threshold [47,49]. Kyoto Encyclopedia of Genes and Genomes (KEGG) (https://www.genome.jp/kegg/ (accessed on 2 February 2021) pathway analysis, WikiPathways analysis (https://www.wikipathways.org/index.php/WikiPathways (accessed on 2 February 2021), and Reactome (https://reactome.org/ (accessed on 2 February 2021) pathway analysis of differentially expressed coding genes was performed. Moreover, Gene Ontology (GO) analysis for biological processes, cellular components, and molecular function [50,51], and CORUM analysis were executed and represented with ClueGO app (http://www.cytoscape.org/ (accessed on 4 February 2021), a Cytoscape plug-in developed to facilitate the biological interpretation and to visualize functionally grouped terms [52]. Moreover, regulatory motifs in DNA with TRANSFAC, miRNAs targets from miRTarBase and human disease phenotypes from Human Phenotype Ontology (HP) analyses were performed [49]. The R software was used to generate heatmaps (heatmap.2 function from the R ggplots package), PCA plot (prcomp function from the R ggplots package), volcano plots [53], and dotplot graphs (ggplot2 library).

### 2.10. RNA Secondary Structure Prediction, Pairwise Alignment, Subcellular Localization, and Functional Annotation

Long non-coding RNAs (RNAs) were selected considering their homology with human GRCh37 genome using MGI web tool (http://www.informatics.jax.org/ (accessed on 4 February 2021) and were subjected to further analysis. RNA secondary structure of selected lncRNAs was predicted using RNA Fold web server (http://rna.tbi.univie.ac.at/cgi-bin/RNAWebSuite/RNAfold.cgi (accessed on 7 February 2021) with default settings. Pairwise alignment was computed using the Geneious software with default settings (Geneious version 2020.1 created by Biomatters. Available from https://www.geneious.com (accessed on 8 February 2021). Subcellular localization was predicted through the LncLocator software [54] while functional annotation was performed on AnnoLnc2 database [55].

### 2.11. Transcription Factors’ Prediction

Transcription Factors (TFs) binding sites were predicted through the Ciiider software. CiiiDER can retrieve promoter sequences from a gene list or use FASTA format sequences and scan for TFs binding sites using supplied position frequency matrices. Ciiider analysis was performed using the mouse GRCm38 genome and the 2020 JASPAR core non-redundant vertebrate matrices [56]. All promoter regions were defined as spanning −1500 bases to +500 bases relative to the transcription start site.

### 2.12. Analysis of Alternative Splicing Isoforms and Functional Consequences

We used the R package “IsoformSwitchAnalyzeR” to analyze individual isoform switches in Nichoid-growth NPCs with respect to standard floating conditions [57,58]. Isoforms were imported from RSEM via the ImportRdata() function [59]. Isoforms were considered differentially switched and retained for further analysis with difference in isoform fraction (dIF) > 0.1 and FDR < 0.05. The functional consequences of switched isoforms were first analyzed for Nonsense-mediated decay (NMD) status, open reading frames (ORF), and intron retention. Moreover, we predicted other biological implications via external tools such as protein-coding potential with CPAT webserver tool [60], intrinsically disordered regions (IDR) via IUPred2A [61] webtool, and signal peptide via SignaIP webtool [62]. The alternative splicing (AS) patterns of switching isoforms were predicted by spliceR [57,63] to include alternative 3′ acceptor sites (A3), alternative 5′ donor sites (A5), exon skipping (ES), mutually exclusive exons (MEE), AS at TF start sites (ATSS), AS at termination site (ATTS), and intron retention (IR).

## 3. Results

### 3.1. NPCs Expanded Inside the Nichoid Show Differences in Non-Coding RNAs Expression

**Figure 1:**
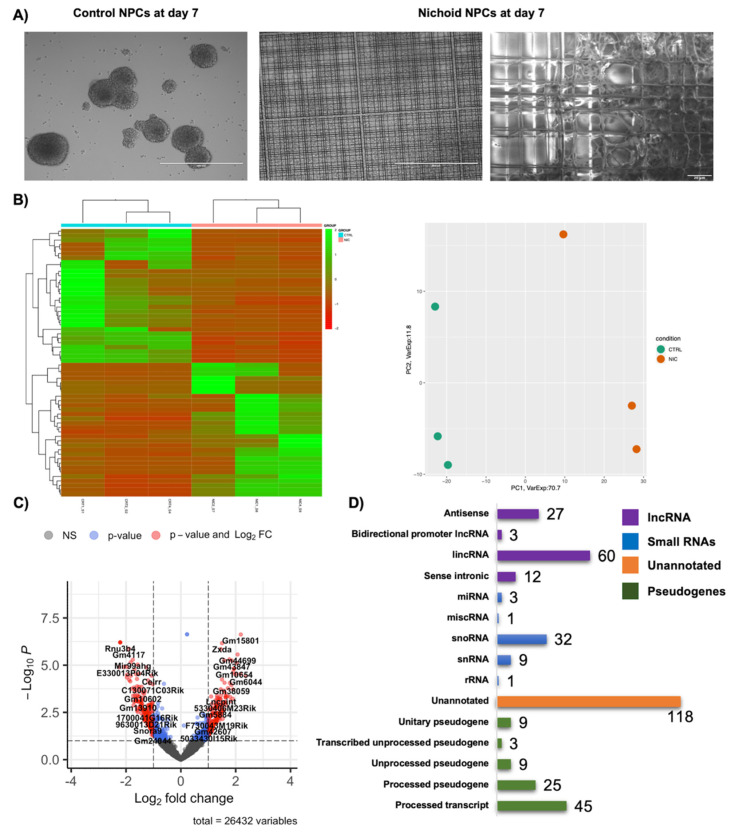
Change in morphology and transcription profiles in Neural Precursors Cells expanded inside the Nichoid. (**A**) On the left, in vivo direct light images (EVOS FL microscope, Euroclone) of NPCs neurospheres maintained in stem cells medium in standard floating conditions (Control NPCs) or grown, with the same medium, inside the Nichoid (Nichoid NPCs) for 7 days. Scale bar 400 μm. Images are representative of observations obtained in more than 10 experiments. On the right, representative images of Nichoid-grown NPCs analyzed by Environmental Scanning Electron Microscope (ESEM). Scale bar 20 μm. (**B**) For RNA-Seq, 3 samples for condition were analyzed. Specifically, three experiments were performed each including one sample of NPCs grown in standard floating conditions for 7 days and one sample of NPCs grown inside the Nichoid for 7 days. The graph shows the Heatmap and the PCA of non-coding differently expressed genes in NPCs grown on the Nichoid and in standard conditions. (**C**) Volcano plot showing only non-coding deregulated genes between NPCs grown on the Nichoid and in standard conditions. (**D**) Bar plot describing the classification of the non-coding deregulated genes found with RNA-Seq approach. Among the 357 non-coding deregulated genes, we identified 118 “unannotated genes”, 102 lncRNAs, 91 pseudogenes, and 46 small ncRNAs.

This work aims at characterizing the deregulation of non-coding RNAs expression in NPCs expanded inside the Nichoid with respect to standard floating conditions. NPCs were grown for seven days both in standard floating conditions [39,41,64] and inside the Nichoid [32]. Nichoid-grown NPCs expanded themselves in the niches showing different morphology to that of the typical spheroids’ conformation (Figure 1A). In particular, cells expanded in standard floating conditions autonomously form neurospheres, while in the scaffold they create a more uniform carpet of cells. This is even more evident from the ESEM images which allow to see in detail the interactions between the 3D scaffold structure and NPCs (Figure 1A). Cells interact directly with the structure and geometry of the scaffold, as can be seen from the presence of cell extensions [32]. Indeed, we demonstrated that over 50% of NPCs grown inside the Nichoid present protrusion in contrast with NPCs grown in standard floating conditions, where the cytoskeleton surrounds cells’ nuclei without protruding from the cell core [32]. This highlights the impact of the Nichoid on cellular morphology and mechanotransduction processes [32]. Thus, from these first qualitative evidences we decided to investigate how the Nichoid exerts its effects on the non-coding transcriptome and we performed a whole transcriptome analysis of NPCs grown in standard floating conditions or inside the Nichoid [32]. Through RNA-Seq we identified 1934 differentially expressed coding and non-coding RNAs (DE RNAs), 1577 were coding genes, whereas 357 were non-coding genes [32]. Heatmap and PCA analysis of the differentially expressed non-coding RNAs (ncDE RNAs) in NPCs expanded inside the Nichoid are shown in Figure 1B and different expression profiles can be visibly distinguished (Figure 1B). The volcano plot here reported highlights the specific non-coding DE RNAs deregulation (Figure 1C). Among the 357 non-coding RNAs that emerged as differentially expressed, 147 were up-regulated and 210 down-regulated. A detailed classification of the functionally present ncDE RNAs is reported in Figure 1D with detailed bar graphs: besides the “unannotated class” represented in orange, which refers to regions that require further experimental validations for the presence of protein coding genes, the most represented functional class is that of lncRNAs represented in violet, with 102 deregulated genes (subdivided in 27 antisense, 3 bidirectional promoter lncRNAs, 60 lincRNAs, and 12 sense intronic), followed by the 91 pseudogenes in green, and lastly 46 small ncRNAs in light blue (Figure 1D).

### 3.2. Co-Expression Analysis of Coding and Non-Coding Transcripts

**Figure 2:**
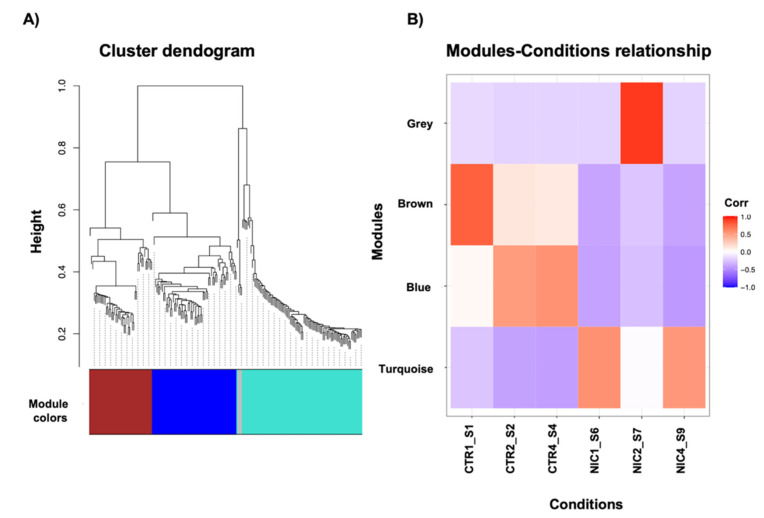
Weighted co-expression analysis network between the top 100 most deregulated coding and non-coding genes in terms of |log2FC|. (**A**) Clustering dendrograms of genes, with dissimilarity based on topological overlap, together with assigned module colors. As a result, 3 co-expression modules were constructed and were shown in different colors. The turquoise module was the largest one with 89 genes, followed by the blue with 62 genes and the brown with 46 genes. (**B**) Heatmap showing correlation between gene modules and conditions (e.g., Nichoid-growth NPCs and standard floating conditions). Different colors are related to different correlation values. For further analysis we focused our attention on turquoise and blue modules.

To investigate the possible interactions between coding [32] and non-coding deregulated RNAs that emerged from RNA-Seq, we constructed a weighted gene co-expression network via WGCNA R package (see M&M section). In Nichoid-grown NPCs and standard floating conditions samples, three gene modules (brown, blue, and turquoise) in total were identified (Figure 2A). Genes which are not co-expressed in any modules are assigned to the grey module. The turquoise module was the largest one with 89 genes, followed by the blue with 62 genes and the brown with 46 genes. For further analysis, we focused our attention on the turquoise and blue modules. According to the Pearson’s correlation coefficient, the turquoise module seems positively correlated with Nichoid-grown NPCs samples (correlation value of 0.5 with the exception for NIC2_S7, where no correlation emerged) and negatively correlated with standard floating condition samples (correlation value of −0.5), as shown in Figure 2B. On the contrary, the blue module seems to be positively correlated with standard floating conditions (correlation value ranging from 0.4 and 0.5) and negatively correlated with Nichoid-grown NPCs (correlation value of −0.7) (Figure 2B).

**Figure 3:**
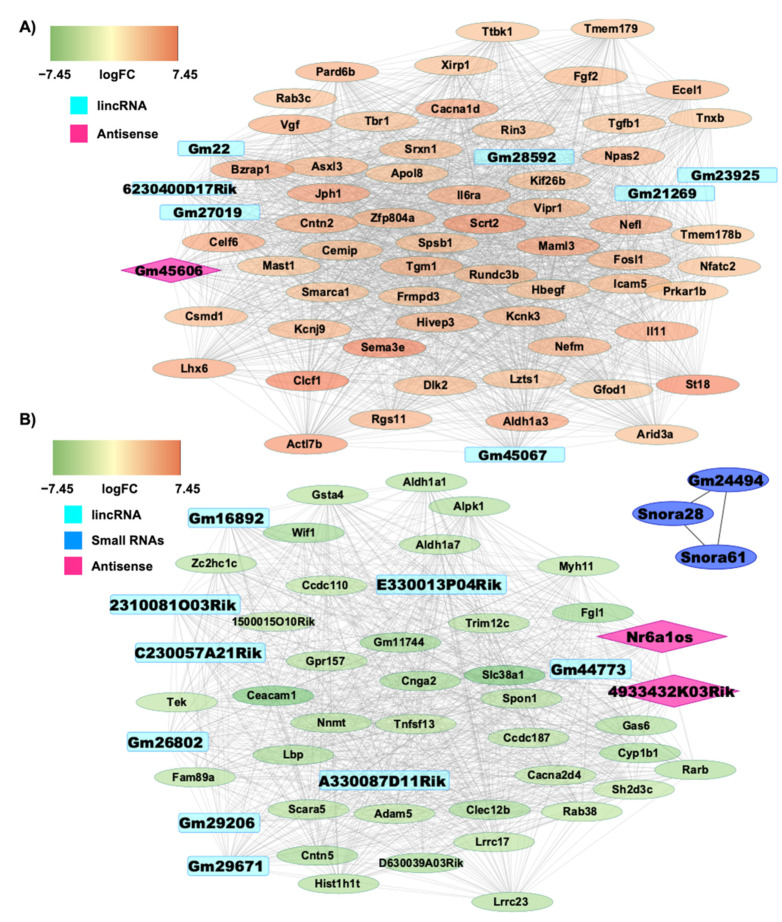
Study of investigated ncRNAs networks. On the basis of the turquoise and blue gene modules, two networks were constructed via Cytoscape. Both networks display coding genes represented in green or orange according to |log_2_FC|, lincRNAs light blue, antisense RNAs in pink, snoRNAs in dark blue. (**A**) The first network was obtained from the turquoise module. Here, 7 lincRNAs and 1 antisense, highlighted respectively in light blue and in pink, interact with each other and with 58 coding genes. All coding genes involved in this interaction are up-regulated and are thus represented in orange according to their fold change. (**B**) The second network was obtained from the blue module. Here, 9 lincRNAs, represented in light blue, and 2 antisense, represented in pink, interact with each other and with 37 coding genes. All coding genes involved in the interaction are down-regulated and are thus represented in green according to their fold change. Moreover, with respect to small ncRNAs, represented in dark blue, *Snora28* co-interacts with *Gm24494* and *Snora61* in a smaller network, forming a non-coding only network.

Furthermore, we constructed interaction networks for these two modules via Cytoscape to highlight the interactions between coding and non-coding genes altered by 3D NPCs expansion. Globally, among the 1577 total coding DE RNAs [32], 92 of them were found to be involved in the interaction with 22 non-coding genes (Table S1). This suggest that ncRNAs could exert their functions through a modulation of these interacting coding DE RNAs. In particular, Figure 3A displays the network built on the basis of the turquoise module. Here, seven lincRNAs (e.g., *6230400D17Rik, Gm28592, Gm22, Gm27019, Gm23925, Gm45067, Gm21269*) and one antisense (e.g., *Gm45606*), highlighted respectively in light blue and in pink in Figure 3A, interact with each other and with 58 coding genes. Interestingly, all coding genes involved in this interaction are up-regulated and are thus represented in orange according to their fold change (Figure 3A). On the other hand, Figure 3B displays the network obtained when considering the blue module. Here, nine lincRNAs (e.g., *Gm44773, Gm29206, Gm26802, Gm26892, 2310081003Rik, Gm29671, A330087D11Rik, C230057A21Rik, E330013P04Rik*), represented in light blue, and two antisense (e.g., *Nr6a1os, 4933432K03Rik*), represented in pink, interact with each other and with 37 coding genes (Figure 3A). Interestingly, all coding genes involved in the interaction are down-regulated and are thus represented in green according to their fold change (Figure 3B). Moreover, with respect to small ncRNAs, represented in blue, *Snora28* co-interacts with *Gm24494* and *Snora61* in a smaller network, forming a non-coding only network (Figure 3B). Remarkably, none of the lncRNAs that emerged in the WGCNA analysis (in light blue or pink respectively in Figure 3), except for *Gm16892* and *Gm28592*, have been functionally characterized yet.

On the basis of the turquoise and blue modules we performed a functional enrichment analysis via the g:Profiler web tool (see M&M section), thus considering not only the different gene modules but also the up and down regulated coding genes that emerged (Figure 3A,B), in order to better investigate the possible functional implications of lncRNAs and small ncRNAs in Nichoid-expanded NPCs. A significant deregulation was observed in all categories analyzed, which include Gene Ontology (GO), KEGG, Reactome, and WikiPathways. Specifically, we identified 184 significant pathways for molecular function (MF), 1428 significant pathways for biological processes (BP), and 130 significant pathways for cellular component (CC). Pathway analyses highlighted 59 significant pathways for KEGG, 151 significant pathways for Reactome, and 16 significant pathways for WikiPathways when considering the turquoise module (Table S2). On the other hand, the down-regulated coding RNAs found in the blue module highlighted 174 significant pathways for MF, 1193 significant pathways for BP, and 73 significant pathways for CC, along with 25 significant pathways for KEGG, 59 significant pathways for Reactome, and 14 significant pathways for WikiPathways (Table S2).

### 3.3. Functional Enrichment Analysis of Genes in Co-Interacting Modules Predicts Functions for Non-Coding RNAs in Gene Ontology

**Figure 4:**
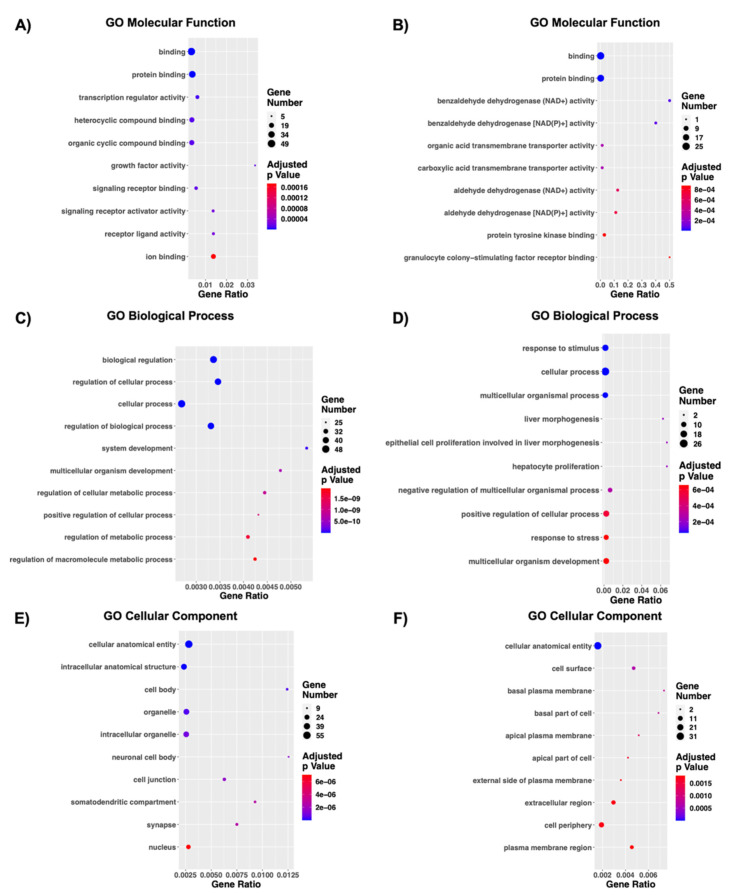
GO enrichment analysis of co-expression modules. On the basis of the turquoise and blue modules we performed a functional enrichment analysis via the g:Profiler web tool by ranking genes according to |log_2_FC|. The top 10 deregulated pathways according to their significance are displayed. A significant deregulation was observed in GO categories, which include BP, MF, and CC. GO MF (**A**,**B**) highlighted respectively 184 significantly deregulated pathways for the turquoise module and 174 for the blue one. GO BP (**C**,**D**) highlighted 1428 pathways emerged as significantly deregulated for the turquoise module and 1193 for the blue one. GO CC (**E**,**F**) highlighted 130 significant terms from turquoise module and 73 form the blue one. The *y*-axis represents the name of the pathway, the *x*-axis represents the Rich factor, dot size represents the number of different genes, and the color indicates the adjusted *p*-value.

The GO Term analysis in MF highlighted 184 significantly deregulated pathways for the turquoise module and 174 for the blue one and the top 10 ranked by significance are displayed as dot-plot graph respectively in Figure 4A,B. Interestingly, the up-regulation following the growth inside the 3D scaffold seems to affect the binding activity. Indeed, when considering the up-regulated coding RNAs that emerged from the turquoise module, 6 out of 10 pathways appear to be related to “binding”, further subdivided into specific binding of protein, ion, signaling receptor, heterocyclic compound, and organ cyclic compound binding (Figure 4A). The term with the highest gene ratio is “growth factor activity”, which suggest a dysregulation of the cell proliferation (Figure 4A). On the other hand, in the blue module, the highest perturbation seems to be due to the down-regulation of two aldehyde dehydrogenase genes: *ALDH1A1* and *ALDH1A7*. Indeed, among the most perturbated pathways it is possible to notice “benzaldehyde dehydrogenase NAD+ activity” “benzaldehyde dehydrogenase NAD(P)+ activity”, “aldehyde dehydrogenase (NAD+) activity”, and “aldehyde dehydrogenase NAD(P)+ activity”, which also present the highest gene ratio (together with granulocyte colony-stimulating factor receptor binding) (Figure 4B). When considering the BP, 1428 pathways emerged as significantly deregulated for the turquoise module and 1193 for the blue one and again the top 10 ranked by significance are displayed as dotplot graph respectively in Figure 4C,D. Both turquoise and blue modules highlighted pathways involved in developmental processes, such as “multicellular organism process”, “system development”, “multicellular organismal development”, and “multicellular organism development”, suggesting a 3D structural organization is key in regulating organism’s development (Figure 4C,D). The regulation of metabolic processes is only correlated with up-regulated genes, whereas the down-regulation in gene expression is related to specific organ morphogenesis (e.g., liver development) (Figure 4C,D).

Lastly, 130 significant terms from turquoise module and 73 from the blue one emerged in GO CC analysis, and the top 10 are displayed as dotplot graph respectively in Figure 4E,F. Both the up- and down-regulated genes are implicated in terms such as “cellular anatomical entity”, “intracellular”, “cytoplasm”, “membrane”, “extracellular region”, and “organelle” highlighting the Nichoid ability to transcriptionally deregulate ncRNAs related to multiple cellular levels, integrating what has been previously reported for coding transcripts [32]. Interestingly, only the up-regulated terms appear to be correlated with neuronal-specific phenotypes, such as “neuronal cell body”, “synapse”, and “somatodendritic compartment” (Figure 4E).

### 3.4. Non-Coding RNAs Modulate Cell Morphology, Signal Transduction, and Cellular Metabolism

**Figure 5:**
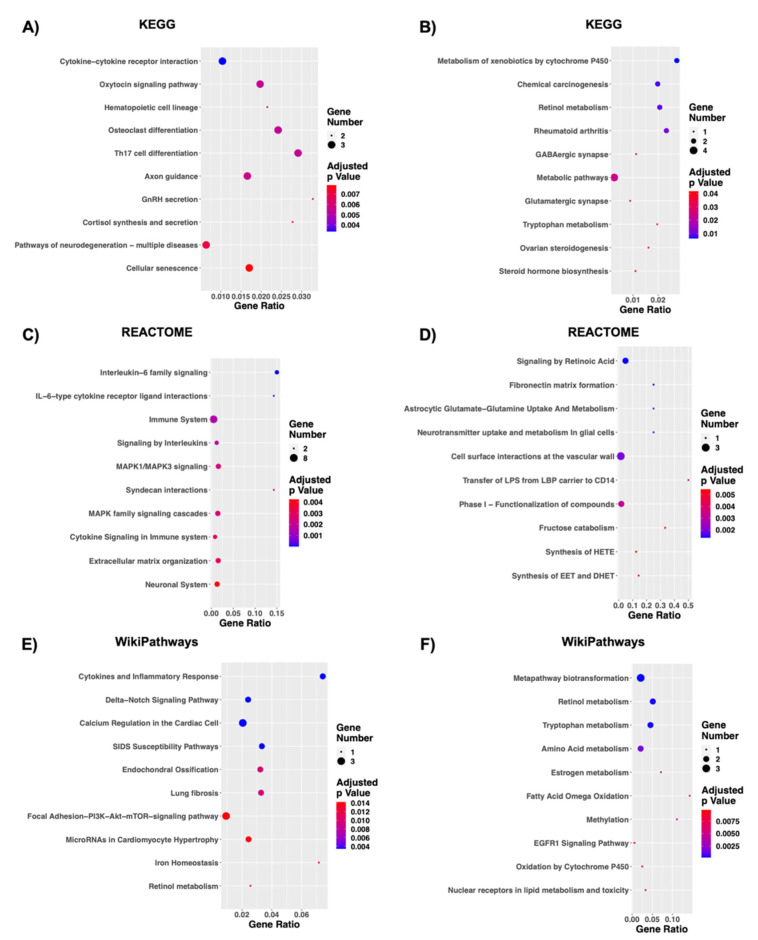
KEGG, Reactome, and WikiPathways enrichment analysis of co-expression modules. On the basis of the turquoise and blue modules we performed a functional enrichment analysis via the g:Profiler web tool by ranking genes according to |log_2_FC|. The top 10 deregulated pathways according to their significance are displayed. A significant deregulation was observed in KEGG, Reactome, and WikiPathways. KEGG (**A**,**B**) highlighted respectively 59 significantly deregulated pathways for the turquoise module and 25 for the blue one. Reactome (**C**,**D**) highlighted 151 pathways emerged as significantly deregulated for the turquoise module and 59 for the blue one. WikiPathways (**E**,**F**) highlighted 16 significant terms from turquoise module and 14 from the blue one. The *y*-axis represents the name of the pathway, the *x*-axis represents the Rich factor, dot size represents the number of different genes, and the color indicates the adjusted *p*-value.

Coding DE RNAs interacting with ncRNAs in the two constructed networks were subjected also to pathway analysis in three well renowned databases: KEGG, REACTOME, and WikiPathways to gain more insights into the biological function and pathway implication taking into account also up- and down-regulation. g:Profiler analysis highlighted 59 significantly deregulated pathways for the turquoise module and 25 for the blue one with KEGG analysis, 151 and 59, respectively, with REACTOME analysis and 16 and 14, respectively, with WikiPathways analysis (Table S2). The top 10 pathways, ranked by significance for turquoise and blue modules are reported respectively in Figure 5A–F. Among the top 10 most deregulated pathways found with KEGG analysis, we identified pathways involved in signal transduction (e.g., “Oxytocin signaling pathway” from the up-regulated terms), synapse alterations (e.g., “GABAergic synapse”, “Glutamatergic synapse”, specifically for down-regulated terms) and metabolism (e.g., “Retinol metabolism”, “Tryptophan metabolism” and “Metabolic pathways” in down-regulated terms) (Figure 5A,B). These findings are supported by REACTOME and WikiPathways analysis. Specifically, the up-regulated coding DE RNAs found in the turquoise module seems to influence mostly signal transduction. Indeed, among the top 10 most deregulated pathways, 5/10 REACTOME deregulated terms and 2/10 WikiPathways deregulated terms were identified as implicated in signal transduction (Figure 5C,E), with a relevant implication for MAPK. On the other hand, the down-regulated genes in blue module are mostly implicated in metabolism, as 3/10 Reactome deregulated terms and 6/10 WikiPathways deregulated terms were identified as implicated in metabolism (Figure 5D,F).

### 3.5. Role of LncRNAs: Focus on Sequence Conservation and Relevance of Human Homologues

Transcriptomic analysis revealed a high number of deregulated lncRNAs (Figure 1D), suggesting that findings concerning mechanotransduction and stemness [30,32] may also be linked to an alteration of these lncRNAs, with a high relevance for them in the regulation of cell fate. Specifically, 102 out of 357 (28%) ncDE RNAs were lncRNAs, and among them, 58% (60 out of 102 lncRNAs) were lincRNAs followed by 26% antisense RNAs (27 out of 102 lncRNAs) (Figure 1D). Moreover, we found five down-regulated lincRNAs (*2900076A07Rik, Gm16892, Gm4262, Gm807, and C130071C03Rik*) associated to pluripotency. *2900076A07Rik, Gm16892, Gm4262*, and *Gm807* activation has been correlated to reprogramming of mouse fibroblasts [65], while *C130071C03Rik* facilitates neuronal differentiation sponging miR-101a-3p in mouse hippocampal tissue [66].

Given the importance of lncRNAs in biomedical research, we focused our attention on those that present human homologues. Specifically, when considering lincRNAs and antisense RNAs, 6 out of 87 had a human homologue, as reported in Table S3. We firstly predicted the subcellular localization for the six lncRNAs via LncLocator web-tool [54] to assess their potential function as it is well-known that lncRNAs localizing in the nucleus are often involved in regulation of gene expression and/or splicing, whereas lncRNAs exported to the cytoplasm can regulate mRNA stability and translation, protein modification, or compete for miRNAs [67]. Interestingly, four out of six lncRNAs with human homologue were predicted to locate inside the nucleus and two of them to the cytoplasm, suggesting that lncRNAs may have an influence in regulating gene expression (Table 2 and Table S4, Figure S1). Then, for each of the six genes with a human homologue (e.g., *Miat, Linc-pint, Mir17hg, Mir679hg, C130071C03Rik, Trp53cor1*), we performed a study of their alignment with the respective human sequence and the prediction of secondary structure based on the minimum free energy (MFE) minimization (Figure S1) predicted according to Turner 2004 RNA folding parameters [68] as it could reveal information about possible interaction partners and function of the transcript. Moreover, genomic location, possible co-expression, and functional annotation for lncRNAs were performed via AnnoLnc2 and are reported in Table 2 and Table S4.

*Miat* is a lncRNA that may constitute a component of the nuclear matrix, located on the negative strand of chromosome 5 and predicted to localize in the nucleus. It presents two different isoforms in mice with six exons each, and four isoforms in humans, two with five exons and two with four exons, with a homology region at the 5′ of the genes (Figure S1). According to the AnnoLnc2 database, it is positively correlated with 228 genes and involved in biological processes like neurogenesis and cell–cell signaling and has molecular functions like binding (Table 2 and Table S4). *Lncpint* places on the negative strand of chromosome 6 and is predicted to localize into the nucleus (Figure S1). It presents three different isoforms in mice, two with four exons and one with three exons, and eight isoforms in humans, four with four exons, one with three exons and three with five exons, with a homology region at the 5′ of the genes (Figure S1). According to the AnnoLnc2 database, it is not correlated to any gene and thus any biological function (Table 2). *Mir17hg* is a lncRNA implicated in cell survival, proliferation, and differentiation that localizes into the nucleus on the positive strand of chromosome 14. It presents only one isoform in mice and two isoforms in humans, one with two and the other one with four exons each (Figure S1). As for *Lncpint*, it is not correlated to any gene and thus to any biological function (Table 2). *Mir670hg* localizes in the cytoplasm on negative strand of chromosome 2 and has nine isoforms in mice and only one little isoform (total length 902) in humans (Figure S1). As for the two previous lncRNAs, it is not correlated to any gene and thus to any biological function (Table 2 and Table S4). *C130071C03Rik* has been found to be involved in neuronal differentiation [66] and places in the cytoplasm along the negative strand of chromosome 13 near other lncRNAs; *C130071C03Rik* also has a few bases in common with its human homologue *LINC00461*. Specifically, it presents four isoforms in mice, two with four exons each, one with three exons, and one with five exons, and 14 isoforms in humans, nine with four exons each, two with five exons each, and three with three exons each (Figure S1). According to the AnnoLnc2 database, it is positively correlated with 79 genes and negatively correlated with two genes (e.g., *Pitpnb* and *Anapc2*). Moreover, it is involved in biological processes involved in neurogenesis (Table 2 and Table S4). *Trp53cor1* is a tumor protein p53 pathway corepressor 1, known also as *linc-RNA p21*. It is predicted to localize into the nucleus on negative strand of chromosome 17; unlike other lncRNAs, it was not possible to carry out the alignment as its sequence in human is not yet defined (Figure S1). According to the AnnoLnc2 database, it is positively correlated with 13 genes and negatively correlated with 16 genes. Moreover, it is predicted to be implicated in biological processes regulating growth and DNA activity (Table 2 and Table S4). A detailed description of lncRNAs with human homologue, as well as the MFE minimization energy predicted is reported in Table 3.

### 3.6. Deregulated LncRNAs Result Associated with Stem Cells Features: Mechanotransduction, Stemness, and Neuronal Differentiation

**Figure 6:**
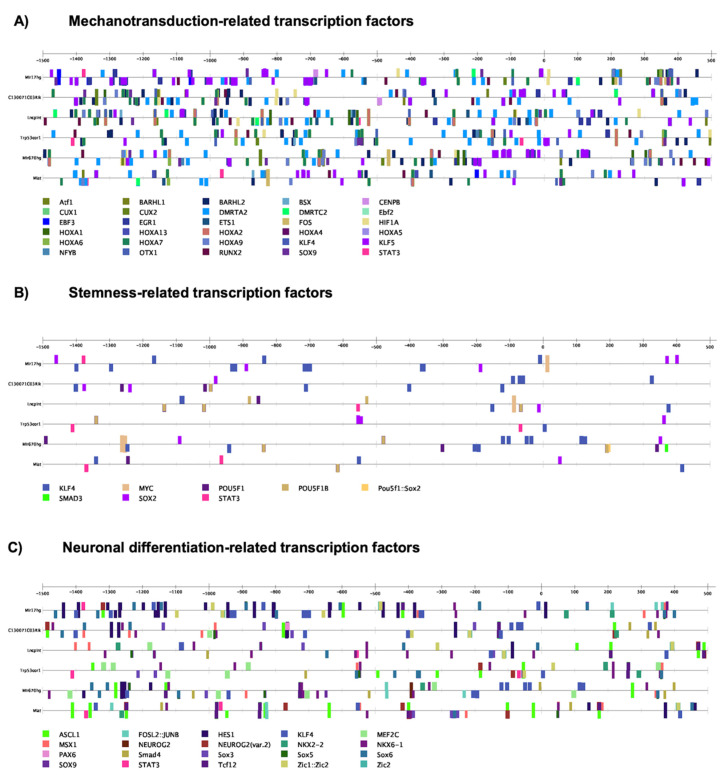
Transcription Factors binding sites for lncRNAs with human homologues. This evaluation was performed with the Ciiider software. Among 644 TFs predicted, 30 out of 644 were associated with mechanotransduction (**A**), 8 out of 644 were stemness TFs (**B**), and 20 out of 644 were neural TFs (**C**).

As already demonstrated, NPCs’ growth inside the Nichoid present altered expression of genes that regulate mechanotransduction and stem cells fate [30,32]. Moreover, as there is a specific alteration of the lncRNAs expression as result of cells’ expansion inside the Nichoid, we investigated whether this could be due to mechanotransduction or stemness-TF regulation. Thus, we predicted the presence of transcription factors binding sites for lncRNAs with human homologue across regulatory region of interest such as promoters or enhancers through the AnnoLnc2 database and the Ciiider software [71], which highlighted 644 TFs. Specifically, among them, 30 out of 644 were associated with mechanotransduction (Figure 6A), 8 with stemness TFs (Figure 6B), and 20 with neural lineage TFs (Figure 6C) [72,73]. When considering TFs related to mechanotransduction, we identified 12 out of 30 TFs involved in cell proliferation, differentiation, and morphogenesis (*KLF4, HOXA5, ATF1, EBF2, EBF3, EGR1, ETS1, FOS, HOXA, KLF5, OTX1, OTX2*) as shown in Figure 6A [74]. *OTX1* and *OTX2* activate genes whose encoded proteins influence the proliferation and differentiation of dopaminergic neuronal progenitor cells during mitosis. Interestingly, among the mechanotransduction-related TFs *EBF2, ETS1, FOS RUNX2, SOX9*, and *STAT3* were predicted also as TFs binding sites for all lncRNAs of interest in the AnnoLnc2 database. Moreover, *Fos1, Fos2,* and *Klf5* were found upregulated in transcriptomic analysis, suggesting that, along with lncRNAs, they could all be part of a common regulatory network that affects proliferation and morphology. Given the importance of the Nichoid in promoting proliferation and stemness potential, we focused our attention also on possible TFs binding sites related to stemness as reported in Figure 6B. In particular, *Myc, Sox2, Oct4,* and *Smad3*, key regulators of pluripotency, emerged also as TFs for these lncRNAs both through the Ciiider software and the AnnoLnc2 database, suggesting that their binding could regulate gene expression with relevance for stemness maintenance.

### 3.7. Identification of Significant Switched Isoforms and Prediction of Alternative Splicing

Our results so far clarify that in addition to canonical phenomena such as gene transcription of the coding genes and therefore of their protein expression, cells’ expansion inside the Nichoid alters other phenomena such as the non-coding epigenome. We then investigated variations in splicing as these can produce differences in gene expression.

**Figure 7:**
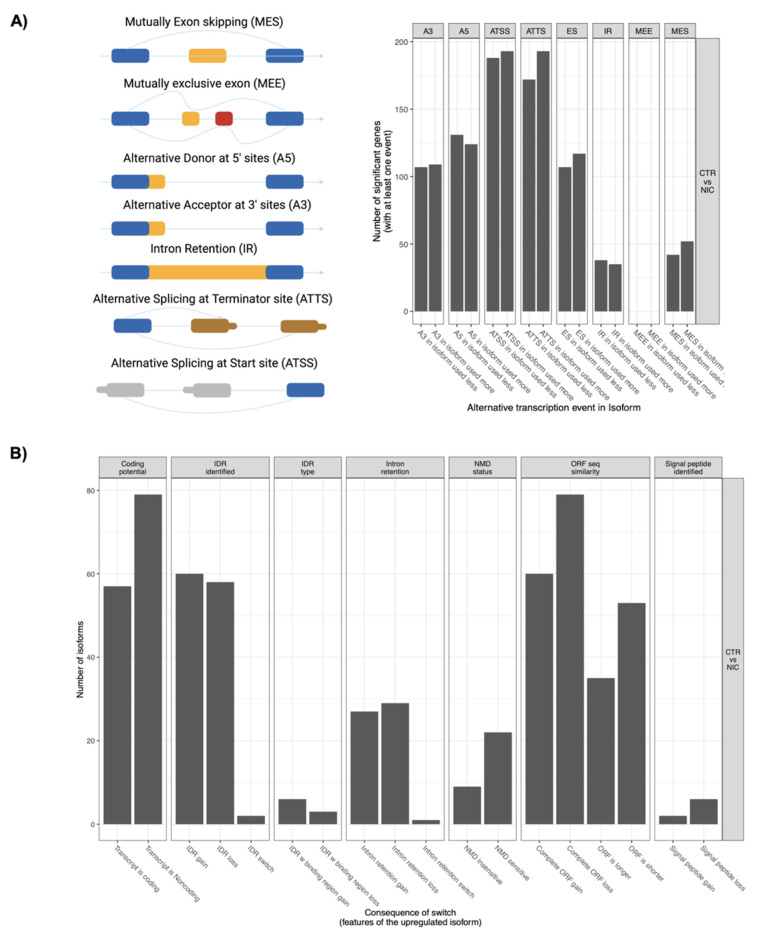

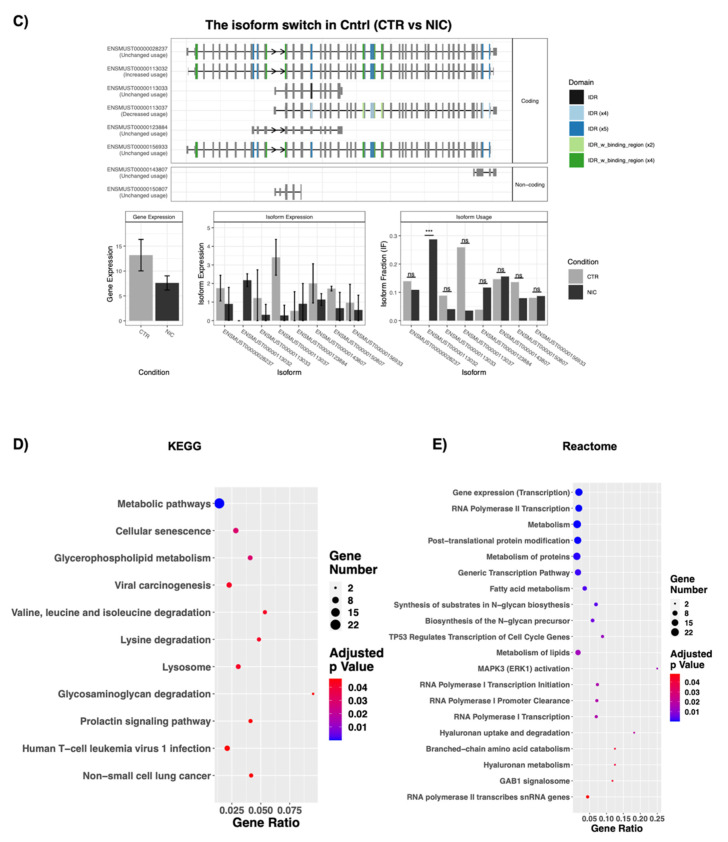
Genome-wide transcripts analysis for switched isoforms between Nichoid-expanded NPCs and standard floating conditions. (**A**) Illustration of alternative splicing event types for the switched isoforms and distribution of isoform usage (increased or decreased dIF) in each type. Created with BioRender.com. (**B**) Overview of the number of switched isoforms predicted to have functional consequences. (**C**) Visualization of switched isoform structure for *Cntrl*. KEGG (**D**) and Reactome (**E**) enrichment analysis for switched isoforms identified. The *y*-axis represents the name of the pathway, the *x*-axis represents the Rich factor, dot size represents the number of different genes, and the color indicates the adjusted *p*-value.

From the expression levels of isoform present when comparing Nichoid-grown NPCs samples and standard floating condition samples, we identified 295 differentially used isoforms with switching features that mapped to 258 unique genes (Table S5). Among these switched isoforms, 178 were protein-coding isoforms and 117 were non-coding isoforms (Table S5). Figure 7A highlights the eight splicing patterns for switched isoform according to different isoform usage when comparing Nichoid-grown samples and standard floating condition samples. Moreover, for each type of splicing, the total number of events detected is reported in Table 4. Some of the switched isoforms are involved in multiple alternative splicing (AS) events (Table S5), observing that the AS events are not equally used (Figure 7A and Table 4). Indeed, ATSS and ATTS are the most predominant with 223 and 213 events respectively followed by A5 (176 events), ES (142 events), A3 (134 events), IR (41 events), and MES (43 events). Furthermore, no MEE events were detected (Table 4).

### 3.8. Analysis of Functional Consequences and Pathways Implication for Switched Isoforms

From the switched isoforms analysis, we further identified 250 significant switched isoforms with predicted functional consequences (Table S5) and the overview of the subsequent biological implications is reported in Figure 7B. The number of IDR identified loss as well as intron retention loss was comparable to IDR identified and intron retention gain, but is much more than switch, where the latter indicates both the gain and the loss. Furthermore, Figure 7C highlights the switched isoform structure of *Cntrl*, which resulted to be the most significant switched isoform with predicted functional consequences (Table S5). Among the eight isoforms that derive from *Cntrl*, ENSMUST00000113032 is the most significantly differentially expressed isoform used in Nichoid-grown NPCs samples. This also corresponds to an increase in isoform expression although overall gene expression for *Cntrl* is decreased (Figure 7C). Lastly, genes involved in isoform switching were subjected to functional enrichment analysis via g:Profiler to evaluate possible biological implications (Figure 7D,E), resulting in 11 KEGG and 20 Reactome terms. For KEGG analysis switched isoforms were mainly associated with different types of metabolic processes, such as “metabolic pathways”, “glycerophospholipid metabolism”, “valine, leucine and isoleucine degradation”, and “lysine degradation”. Reactome analysis showed that switched isoforms were involved not only in metabolism, but also in transcriptional regulation (e.g., “Gene expression”, “RNA polymerase II transcription”, “Generic transcription pathway”, “RNA polymerase I transcription initiation”, “RNA polymerase II transcribes snRNA genes”).

## 4. Discussion

Although the functions of the majority of newly discovered non-coding RNAs are still unknown, some were found to play an important role in the regulation of stem cells [11]. The relationship between 3D cell cultures and non-coding RNAs in the alteration of cellular processes is still unknown. Transcriptional characterization of coding and non-coding genes in 3D scaffolds is of crucial relevance in highlighting new key players and a relevant focus should be placed on non-coding RNAs, as understanding their biological functions still represent one of the major challenges in molecular and cellular biology. Indeed, we have previously reported functional studies extensively describing the biological features of NPCs expanded inside the Nichoid 3D scaffold and their therapeutic efficacy in a preclinical model of Parkinson’s disease [30,32], but a clear annotation of the deregulation in the non-coding epigenome is currently lacking. In this work, we presented a comprehensive analysis of the differential expression of 357 non-coding deregulated genes identified through RNA-Seq in NPCs grown in standard culture conditions and inside the 3D scaffold Nichoid. Deregulated lincRNAs involved in pluripotency, cell survival, and gene expression emerged (*2900076A07Rik, Gm16892, Gm4262, Gm807, C130071C03Rik, Gm26917, Linc-pint, and Linc-p21*). Indeed, *2900076A07Rik, Gm16892, Gm4262,* and *Gm807* activation have been correlated to reprogramming of mouse fibroblasts [66], while *C130071C03Rik* facilitates neuronal differentiation sponging *miR-101a-3p* in mouse hippocampal tissue [67]. The WGCNA co-expression analysis inspected the interaction between coding and non-coding genes. The turquoise and the blue modules highlighted genes with a different trend in term of gene expression. This suggests that different, multiple, and not fully known mechanisms, that should be better investigated, might be key regulators in gene expression [30,32]. Moreover, the identification of co-interaction networks also allowed the identification of numerous targets through which the ncRNAs might exert their functions. Indeed, ncRNAs could possibly influence numerous coding genes found altered via RNA-seq, thus suggesting an involvement in the altered pathways in mechanotransduction, stemness, and neural differentiation that were investigated via KEGG, REACTOME, and WikiPathways, along with GO analysis. The deregulation of pathways related to membrane alteration (e.g., Axon guidance, Extracellular matrix organization, Focal adhesion) as well as cell–cell interaction (e.g., Tight junction) revealed a role for both lncRNAs and small ncRNAs in altering expression of coding genes implicated in mechanotransduction, supporting previous studies [32], but also highlighted their importance in biological processes. Non-coding RNAs were also shown to influence metabolic processes of coding genes, resulting in the overall deregulation of metabolic pathways observed. Indeed, non-coding genes and in particular lncRNAs are emerging as an important class of regulatory molecules controlling the development and function of tissues [75]. The observation that insulin and insulin-like growth factor (*IGF*) 1 signaling also triggers distinct changes in lncRNA expression (e.g., the lncRNA *CRNDE* [76]) points to the fact that lncRNAs may also be implicated in the metabolic effects of insulin and the development of insulin resistance [6]. Thus, a strong interest lies within the identification of lncRNA-mediated mechanisms governing energy and glucose homeostasis at the cell-intrinsic, organ, and whole-body level [76]. Going deeply into the analysis, we found that this response is modulated by the interaction between altered non-coding genes with coding ones. WGCNA co-expression and pathways analysis shed a light on possible mechanisms of interaction between coding and non-coding genes. However, to have a more complete insight of what happens at transcriptome level, it would be interesting to study even more in detail interactions between mRNA-lncRNA-miRNA. Thus, it would be remarkable to take into account the well-known competing endogenous RNAs (ceRNAs) that are groups of non-coding RNAs, mRNAs, and other RNAs that compete with miRNAs at post-transcriptional level, by acting as molecular sponges for miRNAs, thereby regulating mRNAs expression and modulating downstream molecular processes [77]. Lastly, a specific attention was given to lncRNAs in order to evaluate the potential translatability in human stem cells. To this end, we assessed the presence of human homologue sequences of lncRNAs by predicting their subcellular localization, secondary structure, and sequence conservation. Among these, we identified Trpcor51, tumor protein p53 pathway corepressor 1, known also as lincRNA-p21, which has been shown to regulated viability and apoptosis in SH-SY5Y cells treated with MPP+ via targeting α-synuclein, suggesting that *lincRNA-p21* might be a novel target in neurodegeneration, specifically in Parkinson’s Disease [70]. Furthermore, lncRNAs were found to have binding sites at the promoter levels for TFs implicated in mechanotransduction, stemness, and neuronal differentiation, demonstrating how lncRNAs altered expression is strictly correlated to the mechanobiological alterations already explained [30,32]. *Myc*, *Sox2*, *Oct4*, and *Smad3*, key regulators of pluripotency, emerged also as TFs for these lncRNAs both through the Ciiider software and the AnnoLnc2 database, suggesting that their binding could regulate gene expression with relevance for stemness maintenance. Interestingly, *Myc* and *Smad3* were found upregulated in RNA-Seq while *Sox2* and *Oct4* were found upregulated through Real Time PCR [30]. Moreover, in Figure 6C we identified also TFs related to neural differentiation, such as *NEUROG2*, *PAX6*, *NKX6-1*, suggesting also that binding between these TFs and lncRNAs may promote or inhibit the neural differentiation of NPCs. Together these results highlight how lncRNAs could be selectively modulated by specific TFs. Furthermore, we also studied the variation of splicing events as a consequence of 3D cells expansion, with consequent identification of switched isoforms and pathways implication as possible mechanism in altering coding and non-coding gene expression. Among the switched isoforms with functional consequences identified, *Cntrl* emerged as the most significant one. Interestingly, this gene is involved in centrosome maturation and microtubules organization and has been identified as key regulator in asymmetrical division [78], suggesting how splicing events may play an important role in altering gene expression related to cytoskeletal re-arrangement.

## 5. Conclusions

**Figure 8:**
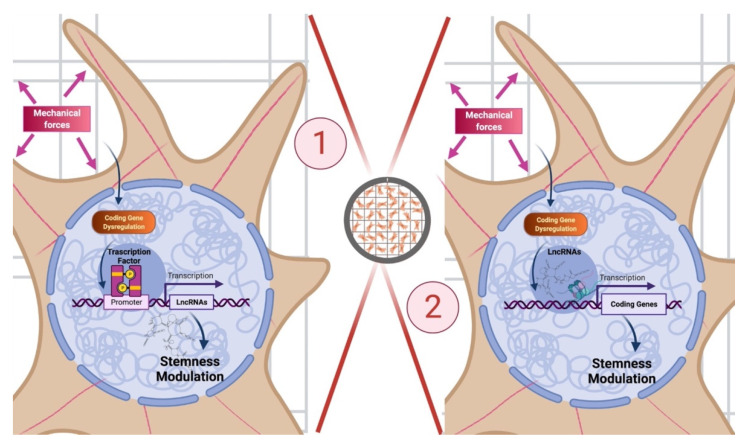
Role of 3D scaffold Nichoid in gene expression and mechanotransduction processes. Enlargement of a cell inside the 3D scaffold Nichoid with two of its possible mechanisms of action. On the **left**, a first proposed mechanism is that mechanotransduction altered by the 3D scaffold might influence coding genes and specific TFs which in turn control non-coding RNAs expression implicated in stemness and pluripotency. On the **right**, a second possible hypothesis is that mecha-notransduction altered by the 3D scaffols might affect non-coding genes that themselves influence coding gene expression and consequently stemness and pluripotency phenotype. Created with BioRender.com.

In conclusion, the results here reported refer to the first analysis of the influence of 3D micro-scaffolds Nichoid on biological and genetic response of non-coding RNAs and alterative splicing events. In particular, the results here presented along with those previously reported [30,32] shed a light on the role of 3D scaffold Nichoid in gene expression and mechanotransduction processes and allow to propose two different hypotheses of how these may work (Figure 8). A first proposed mechanism is that mechanotransduction might influence coding genes and, among them, specific TFs which in turn control non-coding RNAs expression implicated in stemness and pluripotency. A second possible hypothesis is that mechanotransduction might alter non-coding genes that thus themselves influence epigenetically coding gene expression and consequently stemness and pluripotency phenotype. Even if further research and functional experiments are needed to evaluate which of the two is the best hypothesis, the findings here reported demonstrate that the alteration in non-coding genes expression led by the Nichoid strongly affect cell fate, suggesting that non-coding RNAs may be even more crucial than we thought.

## Figures and Tables

**Table 1:** Summary of sequencing reads before and after STAR/RSEM alignment and quantification.

	Number of Input Reads	Uniquely Mapped Reads Number	Number of Reads Mapped to Multiple Loci	Number of Reads Mapped to Too Many Loci
CTR1_S1	19,289,882	11,795,170	1,493,375	37,166
CTR2_S2	23,623,412	14,720,267	1,856,515	38,488
CTR4_S4	23,096,785	14,219,220	1,538,618	25,945
NIC1_S6	13,593,139	90,44,496	833,151	15,608
NIC2_S7	21,106,591	11,528,324	2,345,397	27,963
NIC4_S9	30,114,286	16,176,352	1,197,447	36,228

**Table 2:** Characterization of lncRNAs with human homologues. Genomic location, subcellular localization, the number of positively and negatively correlated genes (co-expression), and the number of functional annotations for lncRNAs with human homologue. Subcellular localization was predicted through the LncLocator tool [54], whereas Genetic location as well as co-expression and functional annotation were obtained through the AnnoLnc2 database [55] (adapted from http://annolnc.gao-lab.org (accessed on 8 July 2021)).

	Miat	Lncpint	Mir17hg	Mir670hg	C130071C03Rik	Trp53cor1
Genetic location	Chr 5, strand −	Chr 6, strand −	Chr 14, strand +	Chr 2, strand −	Chr 13, strand −	Chr 17, strand −
Subcellular localization prediction	Nucleus Score: 0.925	Nucleus Score: 0.987	Nucleus Score: 0.962	Cytoplasm Score: 0.878	Cytoplasm Score: 0.916	Nucleus Score: 0.624
Positively correlated genes	228	-	-	-	79	13
Negatively correlated genes	-	-	-	-	2	16
GO BP	591	-	-	-	360	575
GO MF	127	-	-	-	62	63

**Table 3:** Summary of lncRNAs with a human homologue. Columns report gene name, log2FC, MFE minimization according to the secondary structure prediction and description. Descriptions were taken from literature and ncbi gene bank (https://www.ncbi.nlm.nih.gov/gene/ (accessed on 25 February 2021)).

Gene Name	log2FC	MFE Minimization	Description
*Miat*	2.22	−3699 kcal/mol	This gene encodes a spliced long non-coding RNA that may constitute a component of the nuclear matrix. Altered expression of a similar gene in human has been associated with a susceptibility to myocardial infarction, and is involved in pathways that may contribute to the pathophysiology of schizophrenia.
*Linc-pint*	1.89	−559.80 kcal/mol	lncRNA, Trp53 induced transcript. Its inhibition affects insulin secretion and apoptosis in mouse pancreatic β cells [69].
*Mir17hg*	1.77	−1046.20 kcal/mol	Involved in cell survival, proliferation, differentiation, and angiogenesis. Amplification of this gene has been found in several lymphomas and solid tumors
*Mir670hg*	1.74	−313.30 kcal/mol	RNA Gene affiliated with the lncRNA class. No more information are given
*C130071C03Rik*	−1.12	−997.50 kcal/mol	It facilitates neural differentiation by inhibiting miR-101a-3p’s ability to reduce GSK-3β level [67]
*Trp53cor1*	−1.34	−1075.40 kcal/mol	tumor protein p53 pathway corepressor 1, which is involved also in Parkinson Disease and stemness maintenance [70]

**Table 4:** Summary of detected AS events for switched isoforms according to different isoform usage. For each AS event the total number of events detected is reported.

Type of Splicing Events	Number of Events
ES	142
MEE	0
MES	43
IR	41
A5	176
A3	134
ATSS	223
ATTS	213

## Data Availability

The raw data obtained from the RNA-Seq analysis are deposited on the Gene Expression Omnibus repository (GSE150767).

## Supplementary Materials

The following are available online at https://www.mdpi.com/article/10.3390/biomedicines9091120/s1, Figure S1: Identification of human lncRNAs homologues. For each of the lncRNAs with a human homologue, the figure represents their (A) subcellular localization with LncLocator, their alignment with the respective human sequence, and the prediction of the possible secondary structure based on the MFE minimization. (B) lncRNA Miat, (C) lncRNA Linc-pint, (D) lncRNA Mir17hg, (E) lncRNA Mir679hg, (F) lncRNA C130071C03Rik, and (G) lncRNA Trp53cor1. Table S1: List of coding deregulated genes highlighted by the WGCNA analysis for the turquoise and blue gene modules, Table S2: Functional enrichment analysis of genes resulting from co-expression analysis for the turquoise and the blue gene modules. The table reports the source, the term name and term id, the adjusted *p* value, the term size the intersection size, and the deregulated coding genes involved in the pathways, Table S3: Correlation of lincRNAs and antisense RNAs with human homologues. N/A and No homologue indicates the absence of a respective homologue, conversely the genes which present it are highlighted in bold, Table S4: List of positively and negatively correlated genes and, respectively, functional annotations for lncRNAs with human homologues predicted via AnnoLnc2 database, Table S5: Statistic summaries and functional analysis for significantly switched isoforms between Nichoid-growth samples and standard floating conditions samples, as well as prediction of splicing event patterns for switched genes.
