# Supplementary figures and images for: Neural Precursor Cells Expanded Inside the 3D Micro-Scaffold Nichoid Present Different Non-Coding RNAs Profiles and Transcript Isoforms Expression: Possible Epigenetic Modulation by 3D Growth
Source: Biomedicines. 2021 Aug 31;9(9):1120. doi: 10.3390/biomedicines9091120 (PMC8472193; doi:10.3390/biomedicines9091120)

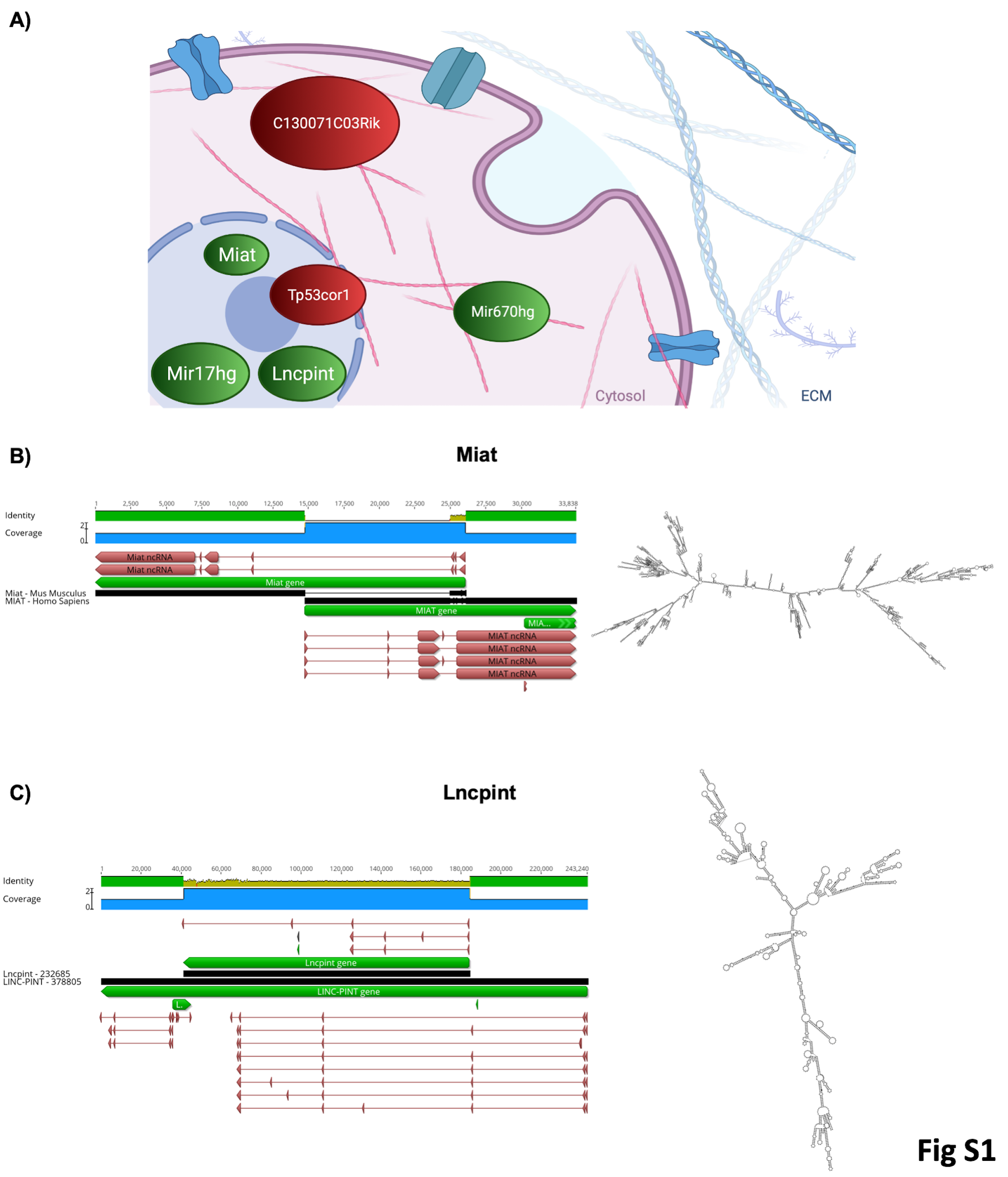

Supplement: Supplementary file 1 [file biomedicines-09-01120-s001.zip › SupplementaryMaterials/FigureS1.tiff]

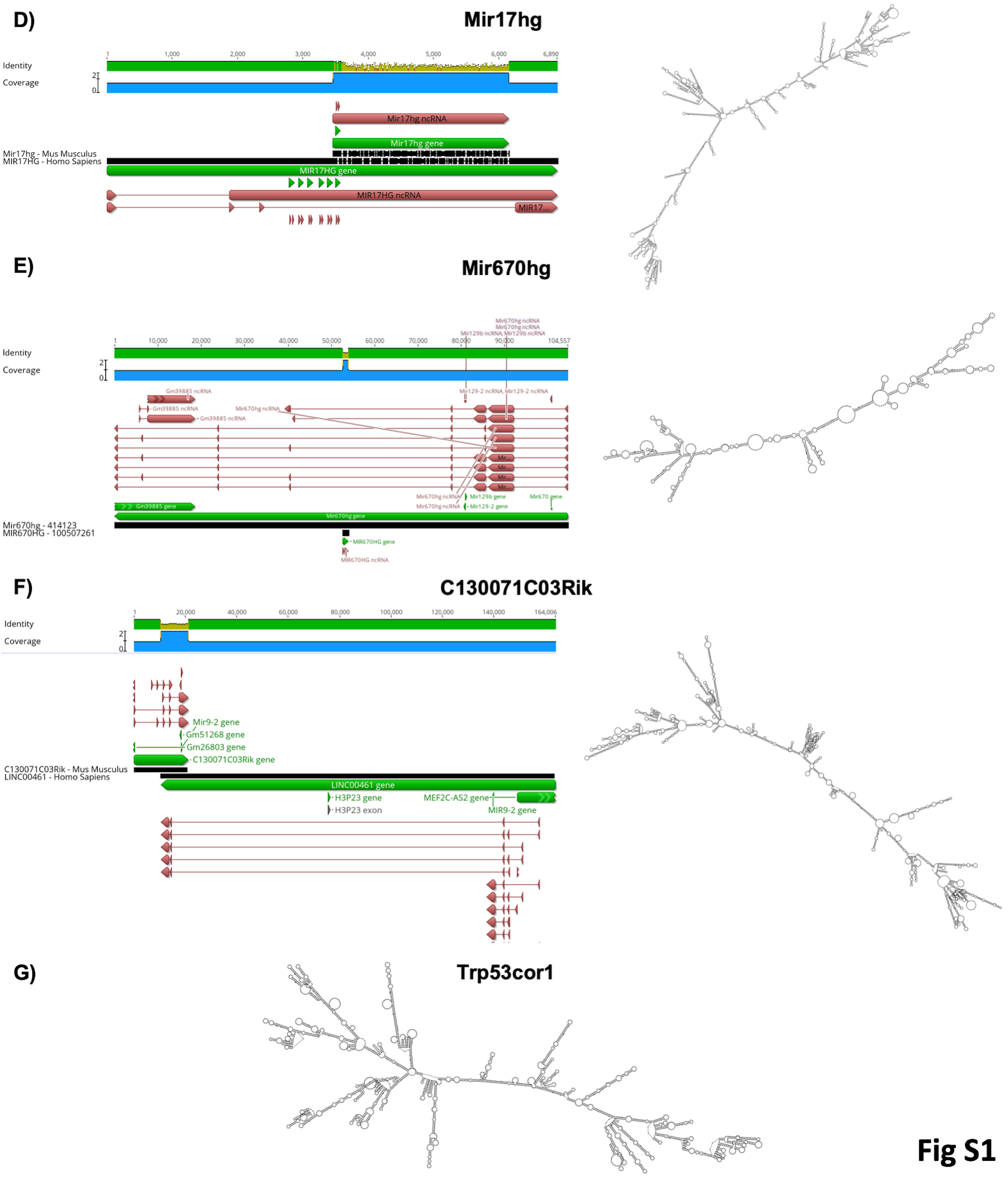

Supplement: Supplementary file 1 [file biomedicines-09-01120-s001.zip › SupplementaryMaterials/FigureS1(2).tiff]
